# Development and Evaluation of Isothermal Amplification Methods for Rapid Detection of Lethal *Amanita* Species

**DOI:** 10.3389/fmicb.2019.01523

**Published:** 2019-07-03

**Authors:** Zhengmi He, Yuting Su, Sainan Li, Pan Long, Ping Zhang, Zuohong Chen

**Affiliations:** College of Life Sciences, Hunan Normal University, Changsha, China

**Keywords:** loop-mediated isothermal amplification, hyperbranched rolling circle amplification, ITS sequence, lethal amanitas, padlock probe

## Abstract

In the present work, loop-mediated isothermal amplification (LAMP) and hyperbranched rolling circle amplification (HRCA) methods were developed to detect and distinguish different lethal *Amanita* species. Specific LAMP primers and HRCA padlock probes for species-specific identification and a set of universal LAMP primers for lethal *Amanita* species were designed and tested. The results indicated that the LAMP-based assay was able to discriminate introclade lethal *Amanita* species but was not able to discriminate intraclade species perfectly, while the HRCA-based assay could discriminate whether introclade or intraclade species. The universal LAMP primers were positive for 10 lethal species of *Amanita* section *Phalloideae* and negative for 16 species of *Amanita* outside section *Phalloideae*. The detection limits of LMAP and HRCA were 10 and 1 pg of genomic DNA per reaction, respectively. In conclusion, the two methods could be rapid, specific, sensitive and low-cost tools for the identification of lethal *Amanita* species.

## Introduction

Mushroom poisoning is the main cause of mortality in food poisoning incidents in China. According to the National Management Information System of Public Health Emergency, in China, 576 mushroom poisoning events were reported from 2004 to 2014, with 3701 poisoning cases and 786 deaths; the fatality rate of mushroom poisoning accounted for 35.57% of total food poisoning (2210) deaths (Zhou et al., 2016). More than 90% of fatal mushroom poisoning cases were caused by mistaken ingestion of lethal amanitas in Europe, North America and East Asia (Enjalbert et al., 2002; Chen et al., 2014). Lethal amanitas are a group of cyclopeptide-containing mushrooms classified in genus *Amanita* section *Phalloideae* (Fr.) Quél. (Cai et al., 2014; Tang et al., 2016). There have been approximately 50 lethal *Amanita* species reported worldwide (Cai et al., 2016). These lethal *Amanita* species have four common morphologic characteristics as bases distinguished from other taxa of *Amanita*, including a non-appendiculate pileus, the persistent presence of an annulus, a bulbous stipe base with a limate volva and amyloid basidiospores (Cai et al., 2014). The containing substances of various peptide toxins were another critical characteristics of lethal amanitas and the peptide toxins in *Amanita* can be divided into three major groups, including amatoxins, phallotoxins and virotoxins, which are bicyclic octapeptides, bicyclic heptapeptides and monocyclic heptapeptides, respectively (Wieland, 1986). The primary toxins responsible for fatal human poisoning of these lethal *Amanita* species are amatoxins that induce acute liver failure through binding with eukaryotic DNA-dependent RNA polymerase II and subsequently inhibiting the elongation essential to transcription (Walton, 2018). In wild, some lethal *Amanita* species are similar to the edible species of section *Caesareae* Singer, for example, *A*. *chepangiana* (edible) vs. *A*. *exitialis* (lethal) and *A*. *hemibapha* (edible) vs. *A*. *subjunquillea* (lethal), this is the main reason for mistaken collection and ingestion.

Rapid identification of poisonous mushroom species eaten by patients is very important for toxic source investigation, clinical diagnosis and proper treatment. Hence, the establishment of rapid and effective methods for detection of lethal amanitas is urgently needed. To date, the identification and detection methods for lethal *Amanita* species mainly depend on their morphological and anatomical evidence, toxin analysis and molecular methods, such as PCR amplification and sequencing of DNA barcoding (Enjalbert et al., 1992; Epis et al., 2010; Harper et al., 2011). However, these methods were often time consuming and complicated and dependent on expensive equipment and professionals, which were difficult to implement in primary institutions or remote areas. Thus, development of simple, rapid and low-cost detection methods would be helpful in curing mushroom poisoning at its early onset as well as investigating the toxin.

In recent years, loop-mediated isothermal amplification (LAMP) and hyperbranched rolling circle amplification (HRCA) have been widely used for molecular detection and identification of pathogenic fungi (Niessen and Vogel, 2010; Tsui et al., 2010; Dai et al., 2012; Davari et al., 2012; Duan et al., 2014; Trilles et al., 2014), in view of their rapid and sensitive detection in addition to the wide range of detection strategies available and the ability of the technique to be deployed outside conventional laboratory settings. LAMP requires a set of four primers (FIP, BIP, F3, and B3) aimed at the six different specific regions of target DNA, and the reaction happens at a constant temperature (60–65°C) catalyzed by *Bst* DNA polymerase (Notomi et al., 2000). A vast number of products (10^9^–10^10^-fold) with a dumbbell structure, which are formed by strand displacement of the outer and inner primers, are produced by cycle amplification. The reaction time is generally about an hour, but if loop primers are added, the time consumed will be shorten by half (Nagamine et al., 2002). Unlike LAMP, the HRCA employs a linear padlock probe that hybridizes with a target DNA and is then ligated by DNA ligase to form a circular probe, which subsequently serves as the template to proceed as a turn-by-turn cascade of multiple hybridization, primer extension, and strand displacement involving two primers under isothermal conditions and finally a >10^9^-fold amplification of products is generated from the reaction (Nilsson et al., 1994; Lizardi et al., 1998).

To date, as far as we know, only one report has been published about the LAMP-based method for rapid mushroom species identification (Vaagt et al., 2013). In this paper, the LAMP assays were used for the rapid and easy detection of the death cap mushroom *Amanita phalloides* from closely related edible and toxic mushroom species. Because there have been many lethal *Amanita* species and similarities among these species, the aims of this study are (i) to develop LAMP and HRCA methods for species-specific identification of lethal *Amanita* species and (ii) to design specific but universal LAMP primers for identification of all lethal *Amanita* species.

## Materials and Methods

### Mushroom Samples and Identification

A total of 26 *Amanita* mushroom species were used in this study, and their information is listed in Table 1, including 10 lethal species in *Amanita* section *Phalloideae* (Supplementary Figure S1) and 16 species of *Amanita* outside section *Phalloideae*. Among them, the *Amanita phalloides* samples were provided by Professor Li TH (Guangdong Institute of Microbiology, China), which were collected from Lazio, Rome, Italy, in October 2014; the *A. bisporigera* samples were collected by Zhang from Hamilton, Canada, in August 2009; and the remaining 24 tested mushroom samples were collected from China. All the mushroom materials were identified by both morphological and molecular evidence (ITS sequence) following Zhang et al. (2010) and Cai et al. (2014). The samples determined in this study were deposited in Mycological Herbarium of Hunan Normal University (MHHNU) and Mycological Herbarium of Guangdong Institute of Microbiology (GDGM).

**Table 1:** Mushroom samples used in this study.

*Amanita* section	Species	Specimen no.	GenBank accession no.
Sect. *Phalloideae*	*A. bisporigera*	MHHNU 7224	KU311692
	*A. exitialis*	MHHNU 30297	KT003192
	*A. fuliginea*	MHHNU 30944	KU356798
	*A. pallidorosea*	MHHNU 8112	KU311697
	*A. phalloides*	GDGM 41101	KT003193
	*A. rimosa*	MHHNU 7954	KU311695
	*A. subfuliginea*	MHHNU 8812	MH142183
	*A. subjunquillea*	MHHNU 7751	KR996715
	*A. subpallidorosea*	MHHNU 8617	KU601411
	*A. virosa*	MHHNU 8621	KY472227
Sect. *Amanita*	*A. rubrovolvata*	MHHNU 8591	KU356797
	*A. rufoferruginea*	MHHNU 30943	KU497532
	*A. sinensis*	MHHNU 8585	KU497533
	*A. sychnopyramis*	MHHNU 30253	KU497534
Sect. *Caesareae*	*A. javanica*	MHHNU 30270	KU497535
Sect. *Vaginatae*	*A. fulva*	MHHNU 8550	KU497536
	*A. orientifulva*	MHHNU 8580	KU497537
	*A. vaginata*	MHHNU 30266	KU497538
Sect. *Amidella*	*A. neoovoidea*	MHHNU 30952	KU497539
Sect. *Lepidella*	*A. kotohiraensis*	MHHNU 30259	KU497540
	*A. oberwinklerana*	MHHNU 30819	KT003191
	*A. pseudoporphyria*	MHHNU 30897	KU497541
Sect. *Validae*	*A. citrina*	MHHNU 30252	KU497542
	*A. orsonii*	MHHNU 8562	KU497543
	*A. sepiacea*	MHHNU 8474	KU497544
	*A. spissacea*	MHHNU 8472	KU497545

### DNA Extraction, PCR Amplification and Sequencing

Total genomic DNA was extracted by the Fungal DNA Mini Kit (OMEGA, United States) and then diluted to 10 ng/μL as a working concentration. The primers ITS 4 and ITS 5 were used for amplification of ITS sequences. The PCR mixtures contained 1 × PCR buffer, 1.5 mM MgCl_2_, 0.2 mM dNTPs, 0.4 μM of each primer, 1.25 U of *Taq* polymerase, and 1 μL DNA template in a total volume of 25 μL. PCR was performed with an Eppendorf Mastercycler thermal cycler (Eppendorf Inc., Germany) as follows: initial denaturation at 94°C for 4 min, followed by 30 cycles of 94°C for 30 s, 54°C for 30 s, 72°C for 30 s, and a final extension at 72°C for 8 min. Amplified PCR products were detected by gel electrophoresis on a 1% agarose gel and then sent to Tsingke Biological Technology (China) for sequencing.

### Phylogenetic Tree Building

Ten ITS sequences of lethal amanitas were obtained by sequencing in this study, and twenty-seven ITS sequences from GenBank were aligned by Clustal X 2.0 software (Larkin et al., 2007). Then, the alignment data of these sequences were used to construct a maximum likelihood phylogeny tree with 1000 bootstrap replicates using MEGA 6.0 software (Tamura et al., 2013).

### Primers and Padlock Probes Design

ITS sequences were chosen as the candidate targets for LAMP primer and padlock probe (PLP) design. For the design, 27 ITS sequences of *A. bisporigera, A. exitialis, A. fuliginea, A. pallidorosea, A. phalloides, A. rimosa, A. subfuliginea, A. subjunquillea, A. subpallidorosea* and *A. virosa* were downloaded from NCBI GenBank and were compared and aligned using DNAMAN 7.0 software to find different target recognition regions for each species and to identify informative nucleotide polymorphic sites conserved within a single species but divergent among different species.

**FIGURE 1:**
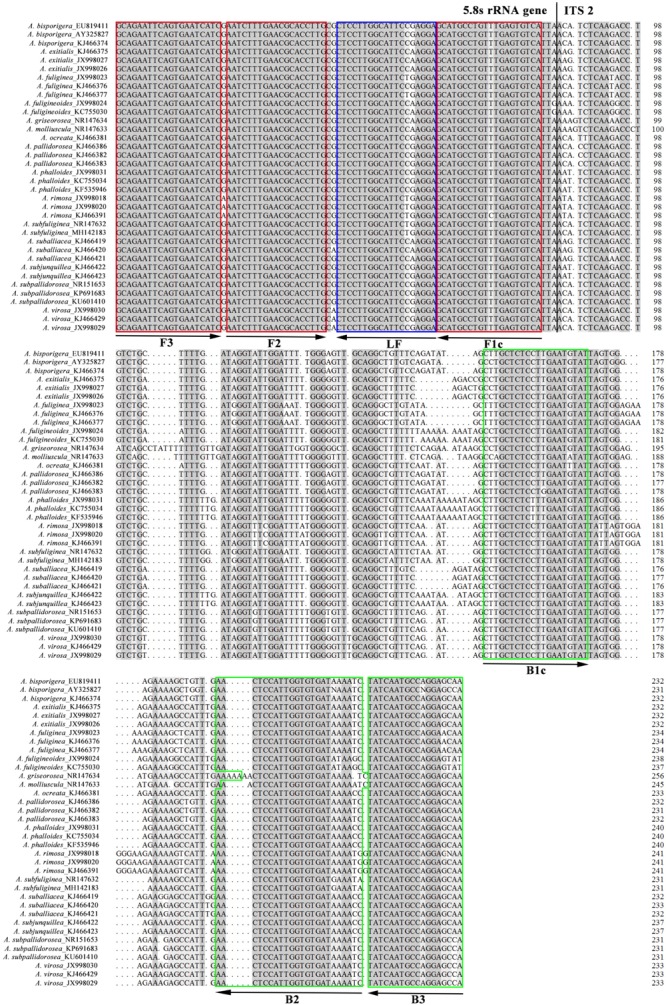
Multiple alignment of ITS sequences of fifteen lethal *Amanita* species. The target regions used for designing the universal primers were labeled with boxes and arrows, and the aligned sequences were partial 5.8S ribosomal RNA gene and internal transcribed spacer 2.

The ten sets of specific LAMP primers were designed by using PrimerExplorer V5^1^. In addition, a set of universal primers for lethal amanitas were manually designed based on the multiple alignment of thirty-six published ITS sequences of fifteen lethal *Amanita* species. A forward inner primer (FIP) consisted of the complementary sequence of F1c and F2, a backward inner primer (BIP) consisted of B1c and B2, two outer primers (F3 and B3). Loop primers (LF or LB) were used for LAMP, and the structure of the universal primers and their complementarity to target DNA are exemplified in Figure 1.

**FIGURE 2:**
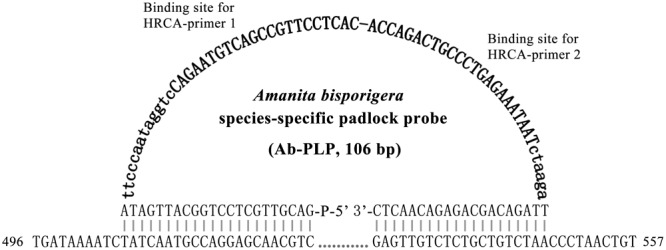
Design of a padlock probe exemplified by the *Amanita bisporigera* probe (Ab-PLP). Linker regions are in bold letters, and HRCA primer binding sites are in bold capital letters. Species-specific sites of the HRCA padlock probe were plain and capitalized. The line of dots indicates connection of adjacent bases, and the numbers 496 and 557 indicate the positions in the ITS region of *A. bisporigera*.

The ten specific PLPs were designed according to criteria as previously described by Kaocharoen et al. (2008) and Lackner et al. (2012). The PLP consists of two terminal regions complementary to a target sequence located at both ends and a linker region in the middle, which was a partial sequence of the inactive X specific transcripts (Xist) gene of *Mus musculus* but lacked homology for the target genes (Figure 2). To ensure the efficiency of padlock probe binding, the padlock probes were predicted with MFOLD to ensure the minimal secondary structure and were designed with the 5′-end probe binding arm Tm (62–66°C) close to or above the ligation temperature (65°C in this study, see below). To increase 3′-end binding specificity, the 3′-end probe binding arm was designed with a Tm (45–48°C) 10–15°C below ligation temperature. The 5′ terminal end of the PLP was modified by phosphorylation to allow ligation. In addition, the HRCA primers (HRCA-primer 1, 2), which are used to amplify the specific padlock probe signal during HRCA, were specifically designed to bind to the flanking linker regions of the above-designed padlock probes (Figure 2).

The primers (PAGE) and PLPs (HPLC) were synthesized by Tsingke Biological Technology (China), and their detailed sequences and lengths are shown in Table 2.

**Table 2:** The information of primers and padlocks used for amplification.

Name	Type	Sequence (5′→3′)	Length (bp)
Ab-primers	F3	GAGGAGCATGCCTGTTTG	18
	B3	GGTCAGACAGTTAGGGTTAG	20
	FIP	CCTGCAACTCCCAAAATCCAtaatGTCATTAACATCTCAAGACCTG	46
	BIP	TTAGTGGAGAAAAGCTGTTGAACTCtataACAGCAGAGACAACTCGACG	49
	LB	AAATCTATCAATGCCAGGAGCAA	23
Ae-primers	F3	AATCTTTGAACGCACCTTG	19
	B3	GACAGTTAGACAGCAGAGA	19
	FIP	ACCCCCAAAATCCAATACCTATCAAtaatGAGCATGCCTGTTTGAGT	47
	BIP	GTGGAGAAAAAGCCATTTGAACTCCtataAACTAGCATTGCTCCTGG	47
	LF	TCAGACAGGTCTTGAGACTTTAATG	25
Af-primers	F3	TGCCTGTTTGAGTGTCATT	19
	B3	GGACATGGATTAGACAGCA	19
	FIP	GCTATACAAGCCCTGCAACCtaatAACATCTCAATACCTGTCTGC	45
	BIP	AGAAAGCTCATTGAACTCCATTGGtataAACTTGGACATTGTTCCTGG	48
	LF	CCCAATTTCCAATACCCATCAA	22
Apa-primers	F3	CATGCCTGTTTGAGTGTC	18
	B3	GTCAAGTGGGTCAGACAG	18
	FIP	GAAACAGCCTGCAACTCCCAtaatATTAACACCTCAAGACCTGTC	45
	BIP	TTAGTGGAGAAAAGCTGTTGAACTCtataGCAGAGACCACTTGATGTT	48
	LB	GATAAAATCTATCAATGCCAGGAGC	25
Aph-primers	F3	GCCTTGCTCTCTTTGAATGT	20
	B3	GATATGCTTAAGTTCAGCGG	20
	FIP	AGTGATATTGCTCCTGGCATTGtaatGAGAAAAGCCATTGAACTCC	46
	BIP	TGCTGTCTAACTGTGACTGTCTtataTAGTCCTACCTGATTTGAGGT	47
	LB	TGGATGGGGACAACTTGACC	20
Ar-primers	F3	TGCCTGTTTGAGTGTCATT	19
	B3	AGTTGGTCAAGTTGTCCAT	19
	FIP	CATTCAAGGAGAGCAAGCTATTTGAtaatGTCTGCTTTTGATAGGTTTCG	50
	BIP	GGTGTGATAAAATGGTATCAATGCCtataCTACAGACAGTTAGCTGAGA	49
	LF	CAACCTGCAACCCCCATAAATC	22
Asubf-primers	F3	TGCCTGTTTGAGTGTCATT	19
	B3	GGGTTAGACAGCAGAGAGA	19
	FIP	GCAAGCCATTTAGAAATAGCCTGCtaatGACCTGTCTGCTTTTGGA	46
	BIP	TTAGTGGAAAAAGCCATTGAACTCCtataGCTGATCATTGCTCCTGG	47
	LF	ACCCCCAAATTCCAATACCCA	21
Asubj-primers	F3	GCCTTGCTCTCCTTGAATGT	20
	B3	GATATGCTTAAGTTCAGCGG	20
	FIP	GTAGTGATATTGCTCCTGGCATtaatGAAAAGCCATTGAACTCCAT	46
	BIP	TGCTGTCTAACTGTGACTGTCTtataTAGTCCTACCTGATTTGAGGT	47
	LB	GGATGGGGACAACTTGACCAAC	22
Asubp-primers	F3	GATTTTTGGGTGTTGCA	17
	B3	AGGTCAAGTTGGTCAAGTT	19
	FIP	CCAATGGAGTTCAATGGCTCTTCtaatGCTTTTTCAGATAGCTTGCT	47
	BIP	TCTATCAATGCCAGGAGCCtataTTACAGACAACTGTGAGA	41
	LB	ATGTTAGTTCTCTCTGCTGTC	21
Av-primers	F3	CATCTCAAGACCTGTCTGTT	20
	B3	AGTTGGTCAAGTTGTCCAT	19
	FIP	GAGTTCAATGGCTCTTTCTCCACTAtaatGGATTTTTGGGGGTTTGC	47
	BIP	TCTATCAATGCCCAGGAGCCtataAGACAACTGTTAGCGGTTAG	44
	LF	CATTCAAGGAGAGCAAGCTATCTG	24
Universal primers	F3	GCAGAATTCAGTGAATCATC	20
	B3	TTGCTCCTGGCATTGATA	18
	FIP	TGACACTCAAACAGGCATGCtaatAATCTTTGAACGCACCTTG	43
	BIP	CTTGCTCTCCTTGAATGTATTAGTtataGATTTTATCACACCAATGGAGTT	51
	LF	TCCTCGGAATGCCAAGGAG	19
Ab-PLP		**P-GACGTTGCTCCTGGCATTGATA**TTCCCAATAGGTCCAGAATGTCAGCCGTTCCT CACACCAGACTGCCCTGAGAAATAATCTAAGA**TTAGACAGCAGAGACAACTC**	106
Ae-PLP		**P-CATTGCTCCTGGCATTGATAGATT**TTCCCAATAGGTCCAGAATGTCAGCCGTTCCTC ACACCAGACTGCCCTGAGAAATAATCTAAGA**GTTAGACAGCAGAGATAACTAG**	110
Af-PLP		**P-CCTGCAACCCCCAATTTCCA**TTCCCAATAGGTCCAGAATGTCAGCCGTTCC TCACACCAGACTGCCCTGAGAAATAATC TAAGA**AGAGCAAAGCTATACAAGC**	103
Apa-PLP		**P-ACTTGATGTTGCTCCTGGCATTGAT**TTCCCAATAGGTCCAGAATGTCAGCCGTTCCT CACACCAGACTGCCCTGAGAAATAATCTAAGA**GGTTAGACAGCAGAGACC**	107
Aph-PLP		**P-TTATTTGAAACAGCCTGCAACCC**TTCCCAATAGGTCCAGAATGTCA GCCGTTCCTCACACCAGACTGCCCTGAGAAATAATCTAAGA**AAGAGAGCAAGGCTATTT**	105
Ar-PLP		**P-TTGTTCATTGCTCCTGGCATTGATA**TTCCCAATAGGTCCAGAATGTCAGCCGTTCCT CACACCAGACTGCCCTGAGAAATAATCTAAGA**ACAGTTAGCTGAGAGAACTG**	109
Asubf-PLP		**P-TCAAGAGACCAGTCAAAAGTCTCTCAT**TTCCCAATAGGTCCAGA ATGTCAGCCGTTCCTCACACCAGACTGCCCTGAGAAATA ATCTAAGA**GATTCCAATTCAAATCAAT**	110
Asubj-PLP		**P-AGTGATATTGCTCCTGGCATTGATA**TTCCCAATAGGTCCAGAATGTCAGCCGTTCCTCACA CCAGACTGCCCTGAGAAATAATCTAAGA**GTTAGACAGCAGAGAGAAGT**	109
Asubp-PLP		**P-GTTAGACAGCAGAGAGAACTAACATGGC**TTCCCAATAGGTCCAG AATGTCAGCCGTTCCTCACACCAGACTGCCCTGAGAAATA ATCTAAGA**TTTTACAGACAACTGTGAGA**	112
Av-PLP		**P-GTTAGACAGCAGAGAGAACTAACATGGC**TTCCCAATAGGTCCAGAATGTCAGCCGTT CCTCACACCAG ACTGCCCTGAGAAATAATCTAAGA**TTACAGACAACTGTTAGCG**	111
HRCA-primer 1		GTGAGGAACGGCTGACATTCTG	22
HRCA-primer 2		ACCAGACTGCCCTGAGAAATAAT	23

*HRCA, hyperbranched rolling circle amplification; PLP, padlock probe; Ab, *A. bisporigera*; Ae, *A. exitialis*; Af, *A. fuliginea*; Apa, *A. pallidorosea*; Aph, *A. phalloides*; Ar, *A. rimosa*; Asubf, *A. subfuliginea*; Asubj, *A. subjunquillea*; Ab, *A. subpallidorosea*; Av, *A. virosa*. taat, tata were the spacers between F1c and F2, B1c and B2, respectively. P represents phosphorylation and bold represents the 5′ and 3′ arms of the PLP matched to the target region.*

### LAMP Reaction and Product Detection

The LAMP reaction was carried out in 10 μL reaction mixtures: 1 × ThermoPol buffer (20 mM Tris–HCl, 10 mM (NH_4_)_2_SO_4_, 10 mM KCl, 2 mM MgSO_4_, 0.1% Triton X-100, PH 8.8), 4 mM MgSO_4_, 1.4 mM dNTP mix, 1.28 μM FIP, 1.28 μM BIP, 0.12 μM F3, 0.12 μM B3, 3.2 U *Bst* DNA polymerase (NEB, United States), 1 μL DNA template (10 ng), 150 μM HNB, and ddH_2_O to 10 μL. The reaction was performed in a 0.2 mL tube with a water bath incubated at 62°C for 60 min and finally 80°C for 10 min to termination.

Two approaches were used to analyze DNA amplification, including direct visual inspection of the color of the LAMP mixture with HNB dye and 2% agarose gel electrophoresis.

### HRCA Reaction and Product Detection

The ligation was carried out in a 10 μL mixture containing: 1 × *Taq* DNA ligase buffer (20 mM Tris–HCl, 25 mM KAc, 10 mM Mg(Ac)_2_, 10 mM DTT, 1 mM NAD, 0.1% Triton X-100), 10 pM linear padlock probe, 12 U of *Taq* DNA ligase (NEB, United States) and 1 μL of DNA template (10 ng). The ligation mixture was incubated at 65°C for 1 h.

After ligation, 1 μL of ligation product was added into an HRCA reaction mixture containing 1 × ThermoPol buffer (20 mM Tris–HCl, 10 mM (NH_4_)_2_SO_4_, 10 mM KCl, 2 mM MgSO_4_, 0.1% Triton X-100), 0.4 mM dNTP mix, 0.5 μM of each HRCA primers and 1.6 U *Bst* DNA polymerase (NEB, United States) with a total 10 μL volume. The reaction was performed in a 0.2 mL tube in a water bath incubated at 62°C for 60 min.

The results were judged by the appearance of color after adding 1 μl of 1000 × SYBR Green I dye to the system after the reaction or 2% agarose gel electrophoresis of the HRCA product.

### Specificity of LAMP Primers and HRCA PLPs

To test the specificity of the ten sets of specific LAMP primers and HRCA PLPs designed above, genomic DNA extracted from *A. bisporigera, A. exitialis, A. fuliginea, A. pallidorosea, A. phalloides, A. rimosa, A. subfuliginea, A. subjunquillea, A. subpallidorosea*, and *A. virosa* was used for cross reaction testing.

For the specificity of the universal primers, genomic DNA from twenty-six *Amanita* species listed in Table 1 was tested by the LAMP method.

### Sensitivity of LAMP and HRCA

To determine the detection limit, the LAMP and HRCA assays were performed using a 10-fold dilution series of genomic DNA from *A. fuliginea* ranging from 10 ng to 10 fg.

## Results

### Specificity of LAMP and HRCA

Genomic DNA from ten lethal *Amanita* mushrooms was used to test the specificity of the corresponding sets of specific LAMP primers and specific HRCA PLPs.

**FIGURE 3:**
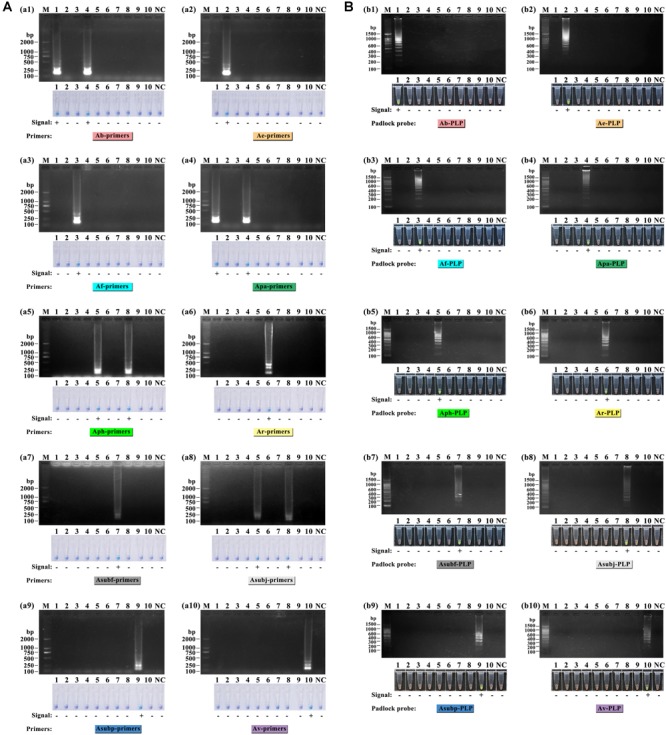
Specificity test of the ten sets of LAMP primers **(A)** and ten HRCA padlock probes **(B)** for lethal amanitas. M: DL2000 or 100 bp ladder, 1: *A. bisporigera*, 2: *A. exitialis*, 3: *A. fuliginea*, 4: *A. pallidorosea*, 5: *A. phalloides*, 6: *A. rimosa*, 7: *A. subfuliginea*, 8: *A. subjunquillea*, 9: *A. subpallidorosea*, 10: *A. virosa*, NC: negative control.

As shown in Figure 3A, the LAMP reactions were analyzed by HNB dye staining and agarose gel electrophoresis. The results of the two detection methods were consistent. Positive reactions were observed with a sky-blue mixture and typical ladder-like banding, whereas for the negative reactions, the color of the tubes remained violet, and no bands were detected after electrophoresis. The six primer sets, Ae-primers, Af-primers Ar-primers, Asubf-primers, Asubp-primers and Av-primers, could clearly recognize and distinguish the expected *Amanita* species. However, cross reaction occurred between the Ab-primers and Apa-primers and the Aph-primers and Asubj-primers.

For the HRCA, amplification products were detected by SYBR Green I dye staining and agarose gel electrophoresis. Positive HRCA results generated a typical ladder-like pattern of fragments increasing in size, comprising the monomer and multimer repeats of the amplified product formed by single and multiple copies of the circularized padlock probe, while negative reactions had a clean background. The HRCA signal was also determined by adding SYBR Green I dye after the reactions; positive reactions turned green while negative reactions remained orange. From Figure 3B, it could be seen that the probes could specifically detect their corresponding targets, and no false-positive reaction was observed. The results from analysis with SYBR Green I dye were compatible with those obtained with electrophoresis.

**FIGURE 4:**
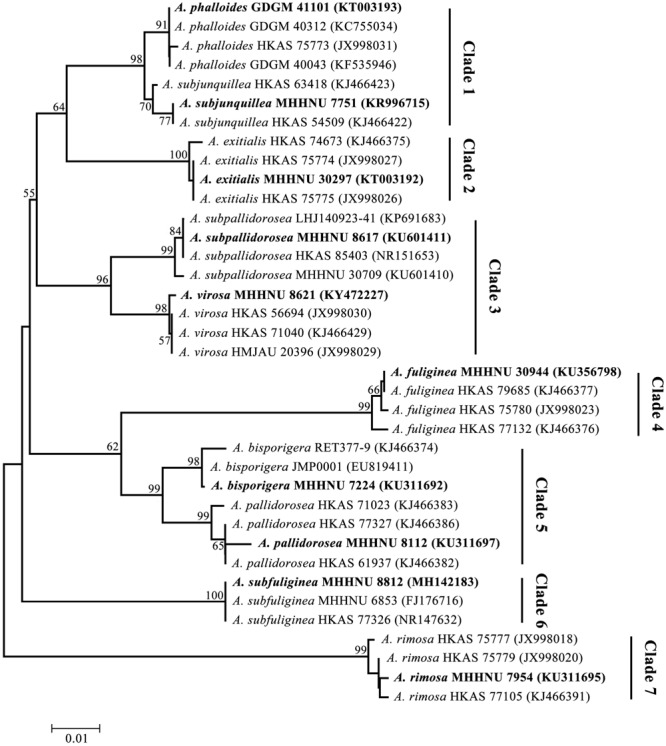
Phylogenetic tree generated from maximum likelihood analysis based on ITS sequences. Bootstrap percentages (>50%) based on 1000 replications are shown at nodes. Bar, a substitution per 100 nucleotides. Sequences in bold were obtained in this study, and the others were from NCBI GenBank.

In addition, the phylogenetic relationship of the lethal *Amanita* species based on ITS sequences was analyzed, and the resulting tree (Figure 4) strongly resolved the examined taxa into seven clades comprising ten phylogenetic species. These results are consistent with the previous results of Cai et al. (2014). *A. exitialis, A. fuliginea, A. rimosa*, and *A. subfuliginea* formed a clade alone with 99 or 100% bootstrap percentages, while *A. bisporigera* and *A. pallidorosea, A. phalloides* and *A. subjunquillea*, and *A. subpallidorosea* and *A. virosa* formed a clade but were classified into two branches with 99, 98, and 96% bootstrap, respectively. By combining the tree and the amplification signals above, it could be intuitively found that LAMP was capable of discriminating interclade lethal *Amanita* species but could not perfectly discriminate the intraclade species (Clade 1 and 5 failed, Clade 3 succeeded); however, HRCA could discriminate intraclade species well. Hence, it could be concluded that the specificity of HRCA was clearly higher than LAMP.

### Evaluation of Universal LAMP Primers

**FIGURE 5:**
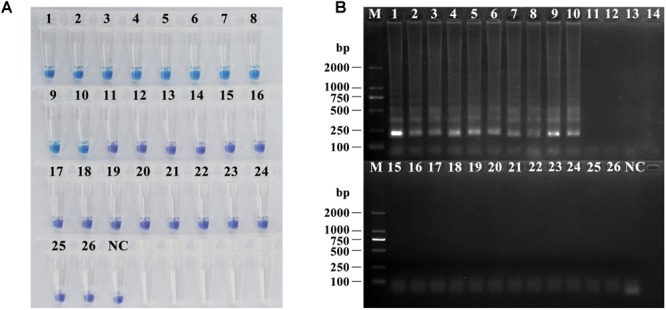
Specificity and universality tests of the universal primers for lethal amanitas. **(A)** Coloration of LAMP by adding HNB dye; **(B)** Electrophoresis analysis of LAMP-amplified products. M: DL2000, 1: *A. bisporigera*, 2: *A. exitialis*, 3: *A. fuliginea*, 4: *A. pallidorosea*, 5: *A. phalloides*, 6: *A. rimosa*, 7: *A. subfuliginea*, 8: *A. subjunquillea*, 9: *A. subpallidorosea*, 10: *A. virosa*, 11: *Amanita rubrovolvata*, 12: *A. rufoferruginea*, 13: *A. sinensis*, 14: *A. sychnopyramis*, 15: *A. javanica*, 16: *A. fulva*, 17: *A. orientifulva*, 18: *A. vaginata*, 19: *A. neoovoidea*, 20: *A. kotohiraensis*, 21: *A. oberwinklerana*, 22: *A. pseudoporphyria*, 23: *A. citrina* 24: *A. orsonii*, 25: *A. sepiacea*, 26: *A. spissacea*, NC: negative control.

To verify the specificity and universality of the universal primers for lethal amanitas, the LAMP reactions were carried out with genomic DNA extracted from 10 lethal species from *Amanita* section *Phalloideae* and 16 species of *Amanita* outside section *Phalloideae*. As shown in Figure 5, the result showed that positive LAMP reaction occurred only in lethal *Amanita* species, while the other species were negative.

### Sensitivity of LAMP and HRCA

**FIGURE 6:**
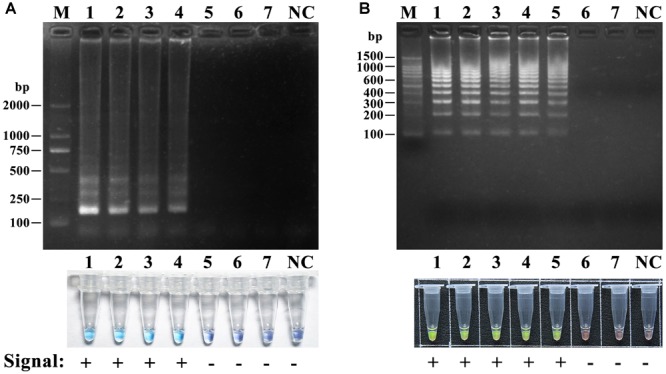
Sensitivity of the LAMP assay **(A)** and the HRCA **(B)** assay for *A. fuliginea*. M: DL 2000; NC, negative control. A dilution series of *A. fuliginea* DNA was as follows: 1, 10 ng; 2, 1 ng; 3, 100 pg, 4, 10 pg; 5, 1 pg; 6, 100 fg; 7, 10 fg.

To determine the detection limit, the LAMP reactions were performed using a serial 10-fold dilution ranging from 10 ng to 10 fg of DNA template of *A. fuliginea*. The detection limit of LAMP and HRCA were 10 pg and 1 pg per reaction, respectively (Figure 6). These results suggested that the detection sensitivity of HRCA was ten times higher than that of LAMP.

## Discussion

In the last 10 years, molecular detection based on ITS sequence has provided a promising alternative strategy for the identification of poisonous *Amanita* species; the ITS sequences could be used as a DNA barcode marker for lethal amanitas (Zhang et al., 2010; Cai et al., 2014). The phylogenetic analysis of the ITS data showed that lethal amanitas (*Amanita* section *Phalloideae*) were robustly supported as a monophyletic group, in which twenty-eight phylogenetic species were divided into nine major clades (Cai et al., 2014). Furthermore, these important molecular characteristics provide a great opportunity for us to design specific or universal primers for the rapid identification of lethal amanitas based on isothermal amplification methods. Vaagt et al. (2013) designed a set of LAMP primers based on the ITS sequence for the specific detection of death cap *A. phalloides*. The limited number of species of *Amanita* in the institute collection did not represent all species of *Amanita*; the related *Amanita* species tested in our study, such as *A. muscaria, A. citrina, A. pantherina*, and *A. rubescens*, are species outside section *Phalloideae*. In our present study, we endeavored to develop a series of species-specific LAMP primers capable of distinguishing each lethal amanitas within section *Phalloideae*. The results showed that the LAMP-based method could distinguish available interclade *Amanita* species but mostly failed to distinguish intraclade *Amanita* species. Some lethal *Amanita* species are very closely evolutionarily related based on small variations in ITS sequences, which are highly similar and identical (Zhang et al., 2010; Cai et al., 2014, 2016). For example, for *A. bisporigera* and *A. pallidorosea*, their ITS are almost the same, with a 98% identity, and they are in the same clade but classified into two branches in the phylogenetic tree (Figure 4), which indicated that LAMP has a certain limitation, and the specificity of the method is not applicable for highly identical templates. Indeed, it was reported that SNP-LAMP was developed to detect allele specific detection or single nucleotide polymorphisms (Fukuta et al., 2006; Ayukawa et al., 2017; Yongkiettrakul et al., 2017). However, it should be noted that some objective factors, such as the base composition of the target template, SNP distribution and amount, melting temperature and GC content of the primer, could affect the final result of SNP-LAMP detection. As reported, Yongkiettrakul et al. (2017) failed to distinguished between the wild-type and quadruple mutant dhfr gene of *Plasmodium falciparum* by SNP-LAMP. In our study, many attempts were made to design SNP-LAMP primers for intraclade *Amanita* species; however, only *A. subpallidorosea* and *A. virosa* (Clade3) were distinguished successfully, and the other two intraclade *Amanita* species, *A. bisporigera* and *A. pallidorosea* (Clade5) and *A. phalloides* and *A. subjunquillea* (Clade1) were not distinguished.

It has been reported hyperbranched rolling cycle amplification coupled with PLP was a particularly useful tool to discriminate closely related species and even subtypes of species with minimal nucleotide polymorphisms (Tong et al., 2007; Najafzadeh et al., 2013; Lin et al., 2018). Therefore, 10 specific PLPs were subsequently designed for each of the 10 lethal amanitas in our present experiment. The results suggested that the HRCA-based assay was able to determine whether each species of 10 lethal amanitas was interclade or intraclade. Notably, *A. bisporigera* was clearly distinguished from *A. pallidorosea*, and *A. phalloides* was also clearly distinguished from *A. subpallidorosea* by HRCA. Even though these two pairs failed to be distinguished by LAMP, these results indicated that HRCA had a higher specificity than LAMP. The high specificity of HRCA resulted from the single base recognition capability of the PLP, which is sensitive to mismatches between the probe and the target (Pickering et al., 2002; Szemes et al., 2005). It was confirmed that mismatches positioned at the 3′ end of PLP were strongly discriminating (Pickering et al., 2002; Szemes et al., 2005), which confers definite and informative target sites for detection. Next, increasing the hybridization temperature and shortening the 3′ arm of the PLP with melting temperature below the ligation temperature are considered to further improve specificity (Faruqi et al., 2001; van Doorn et al., 2007). According these rules, the PLPs designed in our study were preferred with more discriminating bases in the 3′ terminal and short 3′ arms, which induce extremely high specificity.

Furthermore, to distinguish the lethal *Amanita* species in section *Phalloideae* from the other *Amanita* species outside of section *Phalloideae*, a set of universal primers was designed based on the multiple alignment of thirty-six published ITS sequences of fifteen lethal *Amanita* species. The results showed that a positive LAMP reaction occurred only in lethal *Amanita* species, while the rest were negative, which indicated this LAMP method could distinguish the lethal *Amanita* species from the other *Amanita* species outside of section *Phalloideae*. Because these lethal *Amanita* species account for over 90% of all fatal mushroom poisonings worldwide, amatoxins are the common chemical property of these *Amanita* species, which induce acute liver failure (Walton, 2018). In the treatment of clinical poisoning, it is sometimes more important to determine the nature of the species than to determine the accurate species; in this case, this universal LAMP method could be used to rapidly determine whether the species is lethal.

Loop-mediated isothermal amplification and HRCA possess a sensitivity advantage that is 10–100 times higher than conventional PCR (Wang et al., 2012, 2015). Our results showed that the detection limits of the two methods could be at the pg level for the mushroom genomic DNA, and the sensitivity of HRCA was 10 times higher than that of LAMP, which was consistent with Wang et al. (2012). Compared to HRCA, the LAMP detection test was more rapid and simple, where white precipitate was generated by the naked eye within approximately 1 h. However, this technique had a very high risk for contamination, and the precipitate was inconveniently observational. Therefore, hydroxyl naphthol blue (HNB) was used as an indicative dye for the LAMP reaction in this study. When HNB was added before the reaction, a positive reaction will produce large amounts of magnesium pyrophosphate precipitate, thus producing Mg^2+^ and a pH change, and the color of the reaction solution change from violet into blue, so the result is easy to observe, and it does not cause aerosol pollution without opening the tube (Goto et al., 2009). In contrast, HRCA was relatively complicated and needed hours for completion for the extra PLP ligation and exonucleolysis steps. Nevertheless, we proved that exonucleolysis could be omitted because background signals caused by linear probes were almost invisible and insusceptible (data not shown), as was also found by Lackner et al. (2012) and Lin et al. (2018). Thus, the exclusion of an exonuclease reaction shortened the procedure by at least 2 h, and HRCA detection could be completed within 2 h (an hour for PLP cyclization and another hour for amplification) in our study. Despite more reagents and procedures, HRCA is more specific and sensitive than LAMP as described earlier. PCR amplification and sequencing of the ITS is the gold standard for mushroom species identification. However, compared with the two isothermal amplification methods above PCR-based method requires the expensive instrument for thermal cycling and extra time and cost for gel electrophoresis and sequencing and the species identification period using sequencing of ITS usually takes 1–2 working day. But for LAMP and HRCA, the identification only requires a water bath for the reaction and the detection can be completed and judged by dye staining within several hours. Therefore, LAMP and HRCA detection are rapider and require lower cost than PCR.

In conclusion, the LAMP and HRCA-based assays established in this study provided rapid, specific, sensitive and cost-effective tools for the detection and identification of lethal amanitas.

## Data Availability

All datasets generated for this study are included in the manuscript and/or the Supplementary Files.

## Author Contributions

ZC conceived and designed the experiments. ZH, YS, and SL carried out the LAMP and HRCA assay. PL carried out the analysis of ITS DNA sequences of all species and phylogenetic tree building. PZ provided some *Amanita* materials and identified the species. ZH and ZC wrote the manuscript.

## Conflict of Interest Statement

The authors declare that the research was conducted in the absence of any commercial or financial relationships that could be construed as a potential conflict of interest.

## Footnotes

1 http://primerexplorer.jp/lampv5e/index.html

## Supplementary Material

FIGURE S1 Basidiomata of nine lethal *Amanita* species. **(A)** *A. bisporigera* (MHHNU 7224); **(B)** *A. exitialis* (MHHNU 30297); **(C)** *A. fuliginea* (MHHNU 30944); **(D)** *A. pallidorosea* (MHHNU 8112); **(E)** *A. rimosa* (MHHNU 7954); **(F)** *A. subfuliginea* (MHHNU 8812); **(G)** *A. subjunquillea* (MHHNU 7751); **(H)** *A. subpallidorosea* (MHHNU 8617); **(I)** *A. virosa* (MHHNU 8621; photos A, D, E, F, G, and H by PZ; photos B, C, and I by ZC).
