# Supplementary figures and images for: Development and Evaluation of Isothermal Amplification Methods for Rapid Detection of Lethal Amanita Species
Source: Front Microbiol. 2019 Jul 3;10:1523. doi: 10.3389/fmicb.2019.01523 (PMC6626908; doi:10.3389/fmicb.2019.01523)

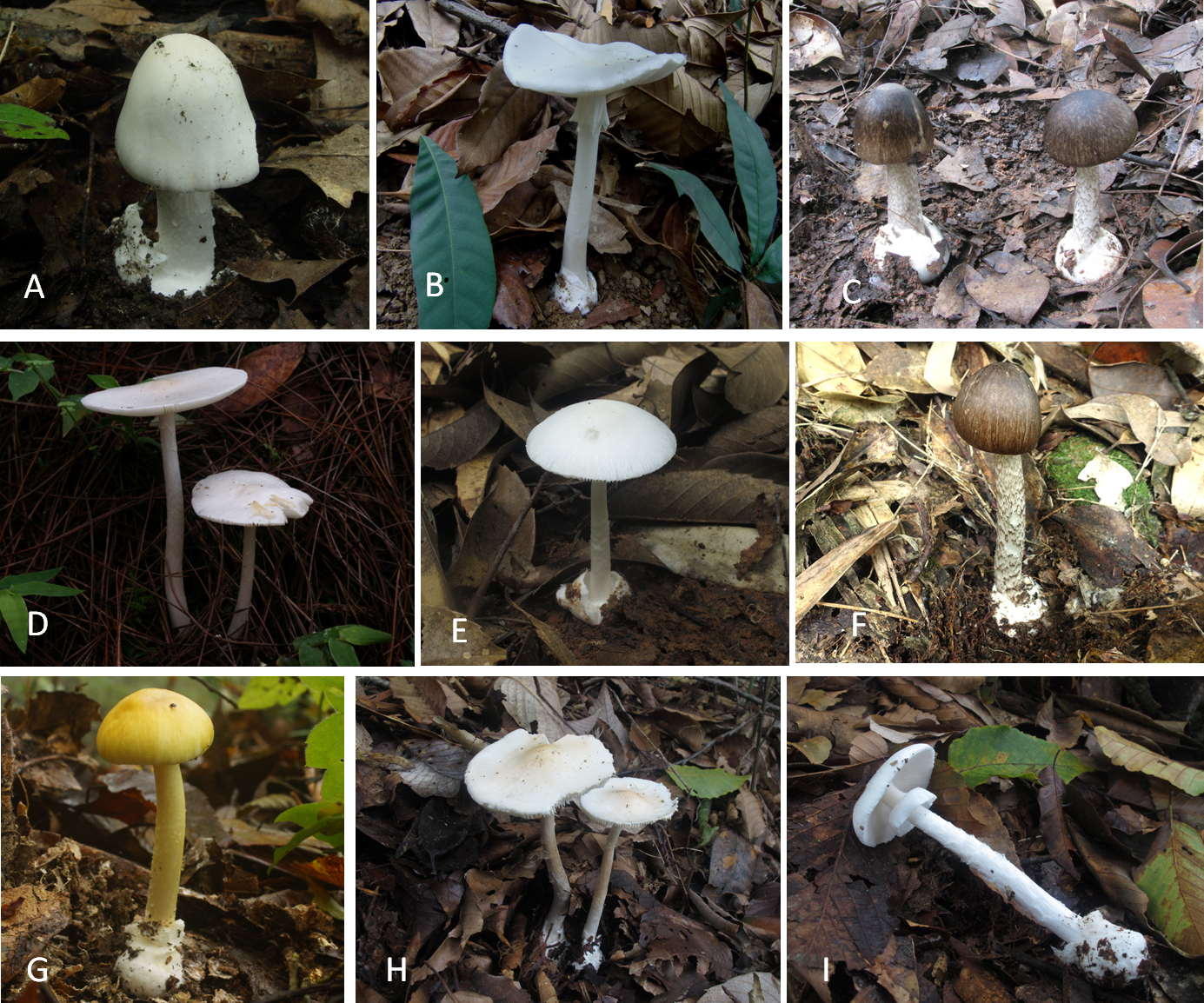

Supplement: FIGURE S1 — Basidiomata of nine lethal Amanita species. (A) A. bisporigera (MHHNU 7224); (B) A. exitialis (MHHNU 30297); (C) A. fuliginea (MHHNU 30944); (D) A. pallidorosea (MHHNU 8112); (E) A. rimosa (MHHNU 7954); (F) A. subfuliginea (MHHNU 8812); (G) A. subjunquillea (MHHNU 7751); (H) A. subpallidorosea (MHHNU 8617); (I) A. virosa (MHHNU 8621; photos A, D, E, F, G, and H by PZ; photos B, C, and I by ZC). [file Image_1.TIF]
